# RNA-Seq Analysis Reveals a Positive Role of HTR2A in Adipogenesis in Yan Yellow Cattle

**DOI:** 10.3390/ijms19061760

**Published:** 2018-06-13

**Authors:** Jinyan Yun, Haiguo Jin, Yang Cao, Lichun Zhang, Yumin Zhao, Xin Jin, Yongsheng Yu

**Affiliations:** 1 Branch of Animal Husbandry, Jilin Academy of Agricultural Sciences, 186 Dongxinghua Road, Gongzhuling 136100, China; yunjinyan@hotmail.com (J.Y.); Khk1962@126.com (H.J.); caoyang003@163.com (Y.C.); zhang_lich@163.com (L.Z.); zhaoym-02-12@vip.163.com (Y.Z.); 2 Agriculture College of Yanbian University, 977 Gongyuan Road, Yanji 133002, China; jinxin@ybu.edu.cn

**Keywords:** adipogenesis, RNA-Seq, DEGs, HTR2A, PI3K/Akt signaling pathway

## Abstract

In this study, we performed high throughput RNA sequencing at the primary bovine preadipocyte (Day-0), mid-differentiation (Day-4), and differentiated adipocyte (Day-9) stages in order to characterize the transcriptional events regulating differentiation and function. The preadipocytes were isolated from subcutaneous fetal bovine adipose tissues and were differentiated into mature adipocytes. The adipogenic characteristics of the adipocytes were detected during various stages of adipogenesis (Day-0, Day-4, and Day-9). We used RNA sequencing (RNA-seq) to investigate a comprehensive transcriptome information of adipocytic differentiation. Compared to the pre-differentiation stage (Day-0), 2510 genes were identified as differentially expressed genes (DEGs) at the mid-differentiation stage (Day-4). We found 2446 DEGs in the mature adipocytic stage relative to the mid-differentiation stage. Some adipogenesis-related transcription factors, CCAAT-enhancer-binding protein α (C/EBPα) and peroxisome proliferator-activated receptor γ (PPARγ) were differentially expressed at Day-0, Day-4, and Day-9. We further investigated the adipogenic function of 5-hydroxytryptamine receptor 2A (HTR2A) in adipogenesis. Overexpression of HTR2A stimulated the differentiation of preadipocytes, and knockdown of HTR2A had opposite effects. Furthermore, functional enrichment analysis of DEGs revealed that the PI3K-Akt signaling pathway was the significantly enriched pathway, and HTR2A regulated adipogenesis by activating or inhibiting phosphorylation of phospho-AKT (Ser473). In summary, the present study provides the first comparative transcription of various periods of adipocytes in cattle, which presents a solid foundation for further study into the molecular mechanism of fat deposition and the improvement of beef quality in cattle.

## 1. Introduction

Beef is a popular meat product worldwide because of religious beliefs, consumer living habits, and excellent meat quality. The content of fat in beef is associated significantly with several meat quality traits. Adipose is a loose connective tissue that consists of adipocytes and functions as the main organ of energy storage and endocrine function in animals, and not only plays an important role in regulating metabolism, but also regulates the amount of fat deposition for animal health and meat quality [1,2]. The fundamental cause of obesity is a long-term imbalance in energy intake and expenditure (i.e., positive energy balance) leading to the increased body mass including the accumulation of subcutaneous and visceral fat [3]. A 5-hydroxytryptamine (5-HT; serotonin) system played an important role in the control of energy homeostasis and glucose homeostasis [4]. Previous studies have shown that serotonin receptors (HTRs) can mediate glycogen synthesis of hepatocytes and participate in the regulation of hepatic glucose metabolism [5]. HTR2A agonist treatment increased lipid accumulation and 5-HT suppressed lipolysis using 3T3-L1 adipocytes [6]. It has been identified as a genetic variant, namely, rs17069005 in HTR2A gene associated with obesity at last follow-up [7], but the role of HTR2A in mediating the effects on cattle adipocyte differentiation remains unexplored.

Beef is a popular meat product worldwide because of religious beliefs, consumer living habits, and excellent meat quality. The content of fat in beef is associated significantly with several meat quality traits. Adipose is a loose connective tissue that consists of adipocytes and functions as the main organ of energy storage and endocrine function in animals, and not only plays an important role in regulating metabolism, but also regulates the amount of fat deposition for animal health and meat quality [1,2]. The fundamental cause of obesity is a long-term imbalance in energy intake and expenditure (i.e., positive energy balance) leading to the increased body mass including the accumulation of subcutaneous and visceral fat [3]. A 5-hydroxytryptamine (5-HT; serotonin) system played an important role in the control of energy homeostasis and glucose homeostasis [4]. Previous studies have shown that serotonin receptors (HTRs) can mediate glycogen synthesis of hepatocytes and participate in the regulation of hepatic glucose metabolism [5]. HTR2A agonist treatment increased lipid accumulation and 5-HT suppressed lipolysis using 3T3-L1 adipocytes [6]. It has been identified as a genetic variant, namely, rs17069005 in HTR2A gene associated with obesity at last follow-up [7], but the role of HTR2A in mediating the effects on cattle adipocyte differentiation remains unexplored.

Increasing amounts of adipocytes caused by cell proliferation and the swelling volume of adipocytes caused by adipogenesis are the main reasons for fat deposits in vivo [8]. Accordingly, fundamentally exploring the potential mechanism of adipogenesis is essential for increasing lipid deposition and improving the quality of beef. Previous studies have demonstrated that the total number of adipocytes may be decided before puberty [9]. For beef cattle, fat cells proliferate within their entire life stages but tend to decrease with age as they tend to be slaughtered early. With increasing age, the progenitor cells of adipocytes gradually decrease, which is also the reason of the decrease in new adipocytes [10,11,12]. The nutritional and physiological feeding styles and developmental period are important for fat production. Transcriptome analysis or gene expression profiling provides a new perspective for the study of molecular mechanisms in adipogenesis [13]. RNA sequencing is a technique for sequencing of mRNA, small RNA, and non-coding RNA (ncRNA) by high-throughput sequencing. The application of NGS (next generation sequencing) makes analyzing the transcriptome and genome of a species possible both sensitively and accurately [12]. Several transcriptome studies involving adipose tissue have been reported using microarray [14] and 3′ digital gene expression-tag profiling [15], and RNA-seq [16].

Although 3T3-L1 is widely used as a commercial cell model of adipocyte differentiation, there exists great diversity between different species, which makes it impossible to determine the adipogenesis and metabolism of actual regulation processes in cattle and other large livestock using mouse adipose cell lines.

In order to determine the importance of differentially expressed genes (DEGs) and pathways in regulation of adipogenesis and the effect of HTR2A on adipocyte differentiation, we isolated Yan Yellow cattle preadipocytes, established a model of adipocyte differentiation, and compared the transcriptome between the preadipocytes and adipocytes by RNA-Seq. We further investigated the adipogenic function of HTR2A in adipogenesis. The transcriptomic data obtained here constitutes invaluable resources for bovine fat deposition and further improves our understanding of the process of adipogenesis. The current study provides the first comparative transcription of various periods of adipocytes in cattle, which presents a solid foundation for further study into the molecular mechanism of fat deposition and the improvement of beef quality in cattle.

## 2. Results

### 2.1. Isolation and Identification of Preadipocytes

**Figure 1:**
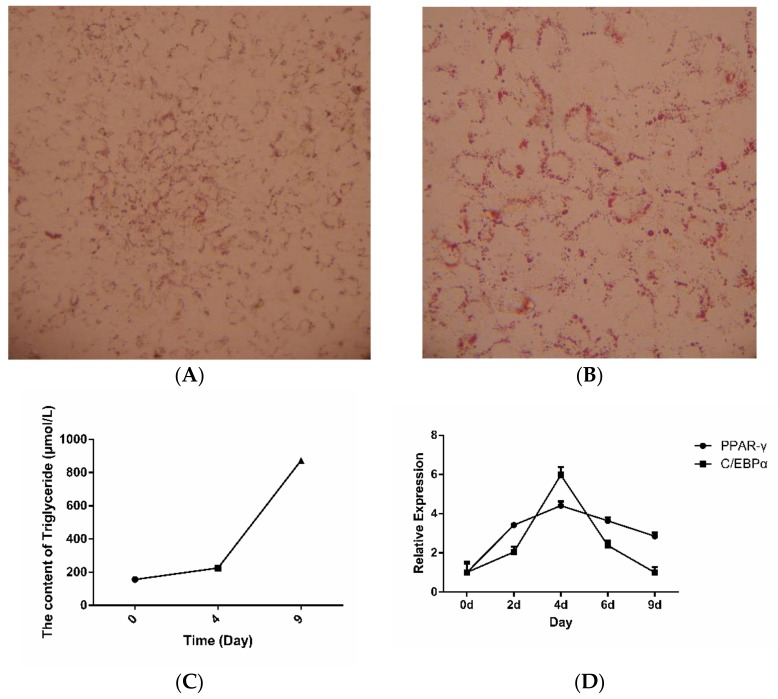
Isolation and identification of pre-adipocytes from Yan Yellow cattle. Undifferentiated cells isolated from Yan Yellow cattle and cultured in medium containing 10% Fetal Bovine Serum (FBS) and stained with Oil-Red O on day 9 post differentiation. (**A**) Identification of the adipocytes with Oil-red O staining, 100×; (**B**) identification of the adipocytes with Oil-red O staining, 200×; (**C**) the contents of the triglycerides were significantly improved with the progression of adipogenesis; (**D**) the relative expression of adipogenic marker genes refers to refers to 2^−ddCt^ with beta-actin as housekeeping control gene.

The accumulated lipid droplet of the adipocytes was stained by Oil red O at Day-9, and the staining results are shown in Figure 1A,B, forming small ring-shaped lipid droplets around the cells. The contents of the triglycerides were significantly improved with the progression of adipogenesis and compared with pre-adipocytes (Figure 1C). These results were also confirmed by Quantitative reverse transcription PCR (RT-qPCR). The expression of adipogenic marker genes changed over the course of induced differentiation of the preadipocytes, with the highest expression levels reached at Day-4 after differentiation induction, and then gradually decreased at Day-9 (Figure 1D). An adipocyte model was established for subsequent successful experimentation.

### 2.2. Transcriptome Library Preparation and Sequencing

The adipocytic transcriptome of Yan Yellow cattle was sequenced using the Illumina HiSeqTM 2500/MiSeqTM platform. We obtained a total of 21,842,223 (total nucleotides: 6.55G), 24,327,149 (total nucleotides: 7.29G), and 22,729,161 (total nucleotides: 6.82G) high quality clean reads (150 bp in length) after removing reads containing poly-N or adaptors and low quality reads from pre-differentiation stage (Day-0), mid-differentiation stage (Day-4), and late-differentiation stage (Day-9), respectively. The Q20 % scores (error identification ratio of nucleotides with Phred quality score < 0.01) of the clean bases were greater than 93% for all these samples, thereby implying high quality sequencing data. The GC content (%) at various stages was 51.52%, 51.77%, and 52.33%, respectively (Table 1 and Figure S1).

Among the sequence reads which passed the quality control, 91.11% in Day-0, 91.61% in Day-4, and 91.08% reads in Day-9 were uniquely mapped to the bovine genome (Bos Taurus. UMD3. 1. 89) using Spliced Transcripts Alignment to a Reference(STAR) (2.5.2a) (Table 2). The results of the STAR alignment were assembled by StringTie (1.2.4). Gffcompare (0.9.7) was used to compare assembled transcripts to the reference genome and identify putative novel transcripts or novel exons of known genes.

### 2.3. Analysis and Identification of DEGs

**Figure 2:**
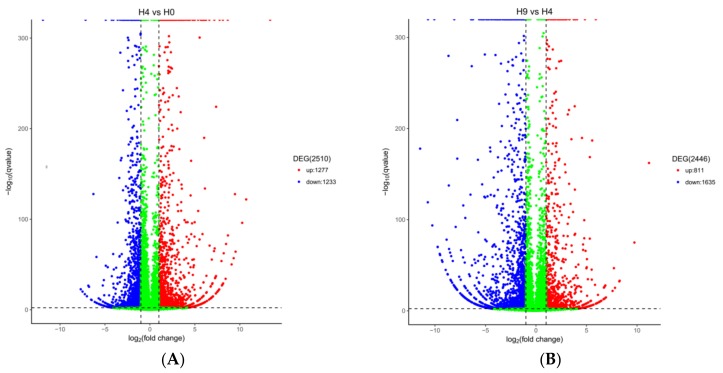

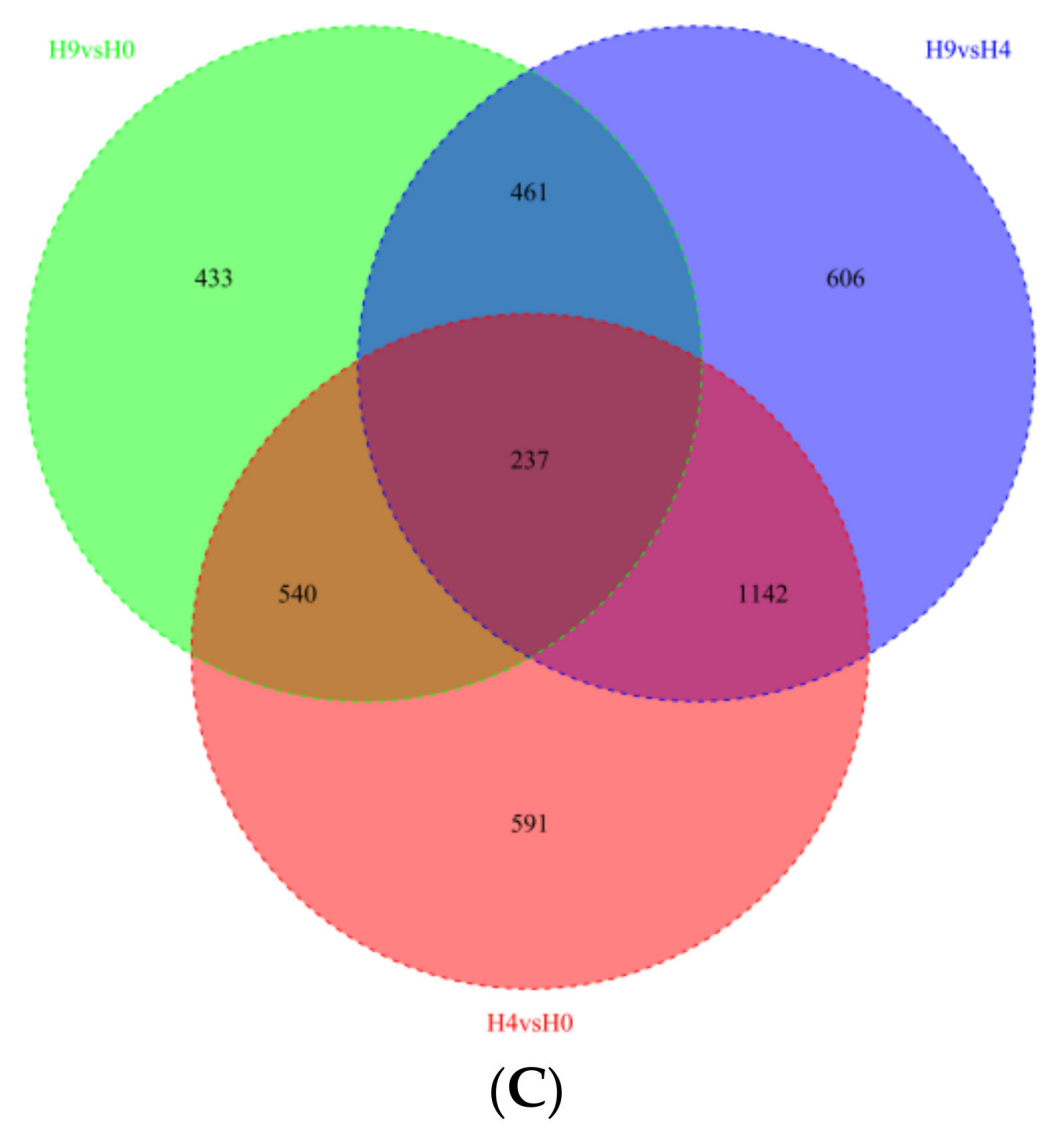
Comparison of differentially expressed genes identified during several adipogenesis stages. (**A**) Volcano Plot analysis of differentially expressed genes (DEGs) in the early stages of adipogenesis; (**B**) volcano Plots analysis of DEGs in the later stage of adipogenesis; (**C**) volcano Plots analysis of DEGs in various stages of adipogenesis.

**Figure 3:**
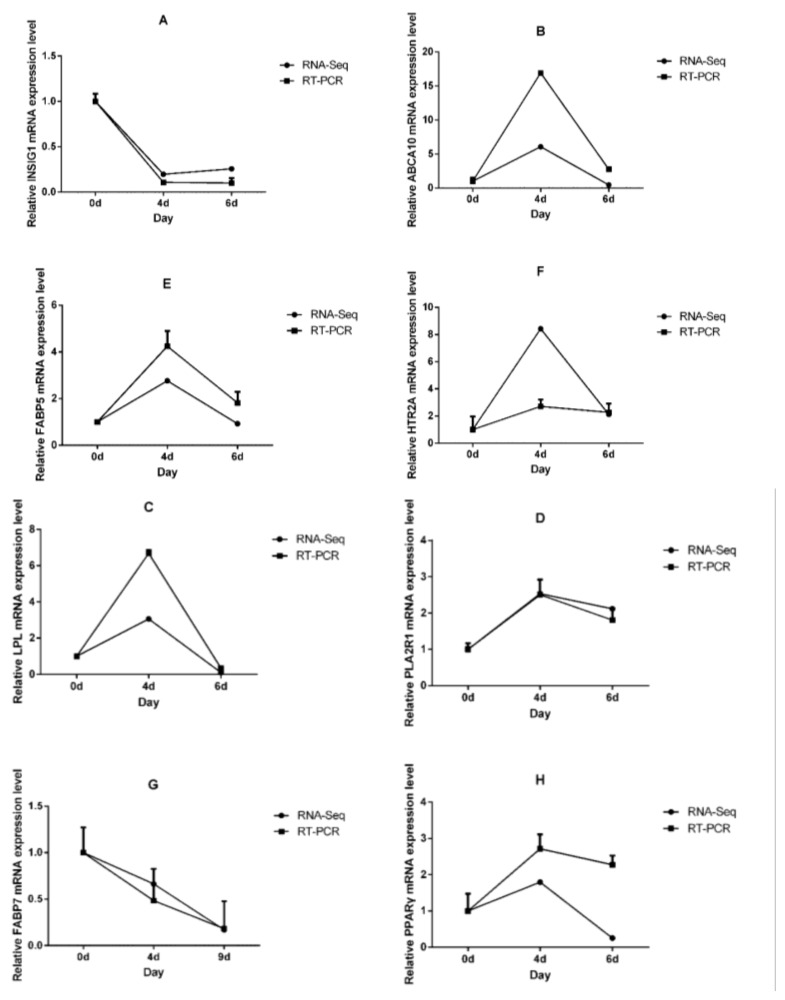
RT-qPCR validation of the DEGs from RNA sequencing. (**A**): Insulin induced gene 1 (INSIG1); (**B**): ATP binding cassette subfamily A member 10 (ABCA10); (**C**): Lipoprotein Lipase (LPL); (**D**): Phospholipase A2 receptor 1 (PLA2R1); (**E**): Fatty acid binding protein 5 (FABP5); (**F**):5-hydroxytryptamine receptor 2A (HTR2A); (**H**): Fatty acid binding protein 7, (FABP7); (**G**): peroxisome proliferator activated receptor gamma (PPARγ); β-actin was chosen as the internal control genes. Each experiment was carried out in triplicate. The solid black circle and square represented differential expression multiple from sequencing and RT-qPCR.

After normalizing read count data with trimmed mean of M-values normalization method (TMM) and setting the significance and fold-change (*p* < 0.05 and log2 fold change > |1|), DEGseq was used for differential expression analysis. We identified 2510 DEGs (including 388 putative novel genes), which included 1277 up-regulated genes and 1233 down-regulated genes on Day-4 compared to Day-0 (Figure 2A); and 2446 DEGs (including 354 novel genes), which included 811 up-regulated genes and 1635 down-regulated genes on Day-9 compared to Day-4 (Figure 2B). When these data were analyzed in combination, 237 genes were differentially expressed during several stages of adipocytic differentiation (Figure 2C). In order to validate these DEGs, we compared the fold change of adipogenesis specific genes in RNA-Seq with those in the RT-qPCR data, for example, Insulin induced gene 1 (INSIG1), ATP binding cassette subfamily A member 10 (ABCA10), Lipoprotein Lipase (LPL), Phospholipase A2 receptor 1 (PLA2R1), Fatty acid binding protein 5 (FABP5), 5-hydroxytryptamine receptor 2A (HTR2A), Fatty acid binding protein 7 (FABP7), and peroxisome proliferator activated receptor gamma (PPARγ). The results demonstrated that the expression levels of RT-qPCR were consistent with the RNA-seq. These results confirmed that the DEGs identified in this study were very reliable (Figure 3). 

### 2.4. Functional Enrichment Analysis of Differentially Expressed Genes

**Figure 4:**
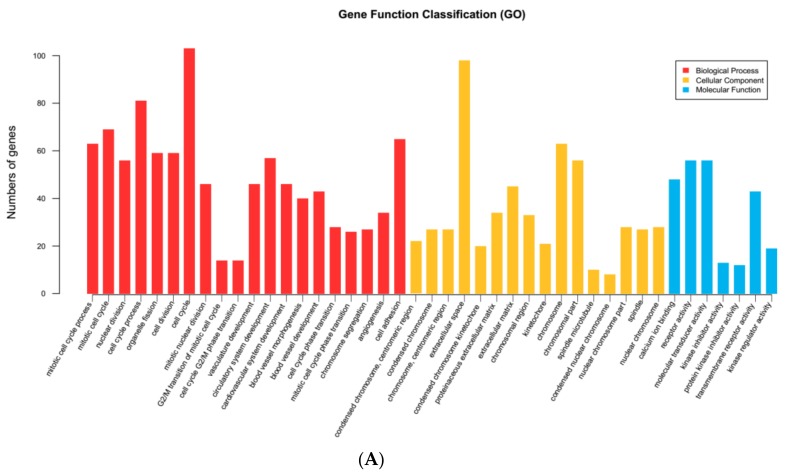

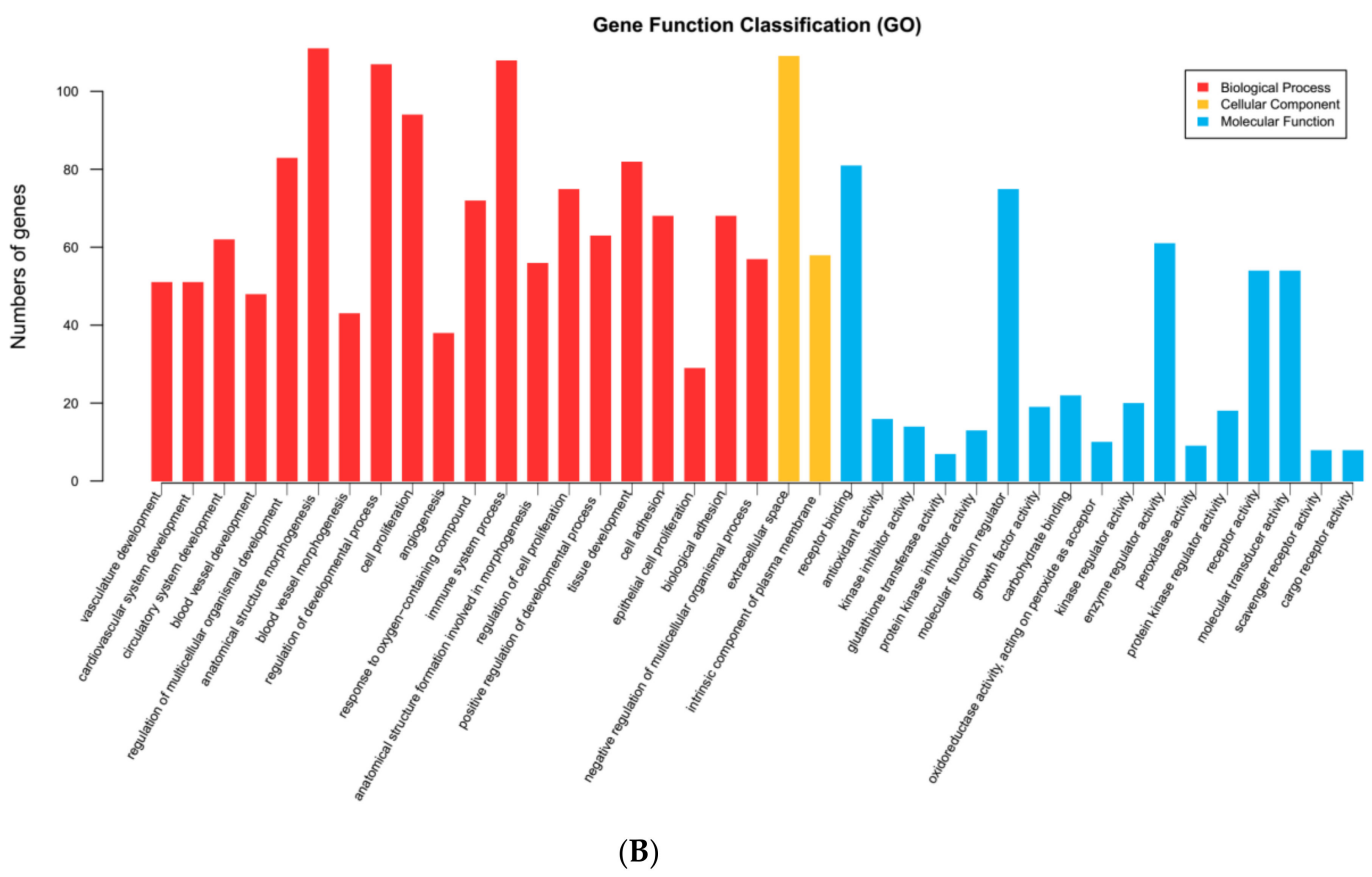
Gene Ontology (GO) annotations of unigenes in the Yan Yellow cattle transcriptome. The figure is composed of three parts: biological processes; molecular functions; and cellular components. The significance level of enrichment was set at a corrected *p*-value (*p* adjust < 0.05). (**A**) GO annotations of unigenes in the early stage of adipogenesis; (**B**) GO annotations of unigenes in the later stage of adipogenesis.

**Figure 5:**
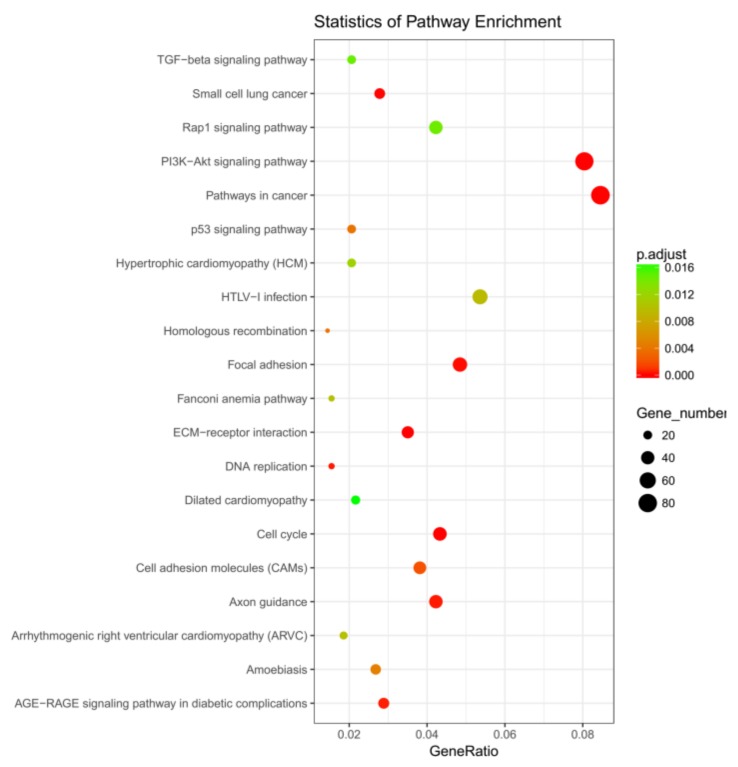

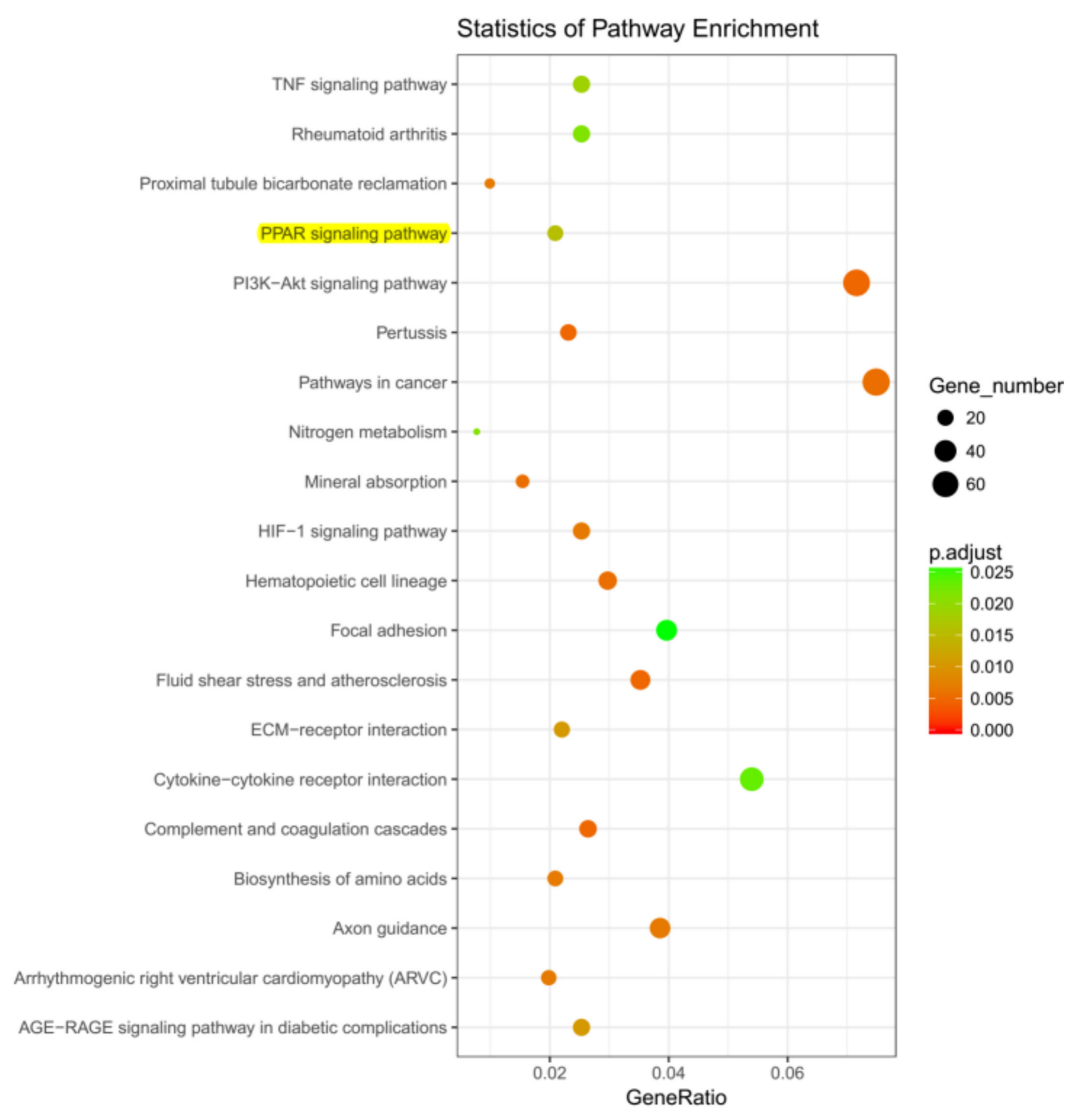
Kyoto Encyclopedia of Genes and genomes (KEGG) functional annotations of unigenes in the Yan Yellow cattle transcriptome. The scatter diagram shows enrichment of DEGs in the signaling pathways. The X-axis label represents GeneRatio (GeneRatio = amount of DEGs enriched in the pathway/amount of all genes in annotation gene set), and the Y-axis label represents the pathway (*p* adjust < 0.05).

To obtain insight into the biological relationship of differentially expressed genes in adipocytes at Day-0, Day-4, and Day-9, after setting the significance threshold (*p* < 0.05), we performed Gene Ontology (GO) and Kyoto Encyclopedia of Genes and genomes (KEGG) pathway enrichment analysis of DEGs using cluster profiler software (3.2.9). Several unigenes were assigned to GO categories with more than 2000 unique functional terms. A GO enrichment histogram reflected directly the quantity distribution of DEGs in biological processes, cellular components, and molecular functions of the GO term (Figure 4A,B). In the biological processes category, the majority of unigenes were associated with cell cycle and cell cycle processes. Within the cellular component category, most unigenes were assigned to chromosomes, extracellular spaces, and chromosomal parts. In the molecular function category, most unigenes were associated with DNA binding and receptor activity. A scatter diagram represented graphically the representation of the KEGG enrichment analysis results, and measured the level of KEGG enrichment by GeneRatio, and the number of genes enriched in this pathway (Figure 5). More than 2200 unigenes were enriched into about 300 KEGG pathways; ECM-receptor interactions, PI3K-Akt signaling pathways, with pathways involved in cancer the most enriched pathways during adipocytic differentiation (Table 3, Figure S1). The PI3K-Akt signaling pathway was the most reported pathway which affected the differentiation of adipocytes and DEGs during various stages and is shown in Table 4.

### 2.5. The Role of HTR2A in the Regulation of Adipogenesis

**Figure 6:**
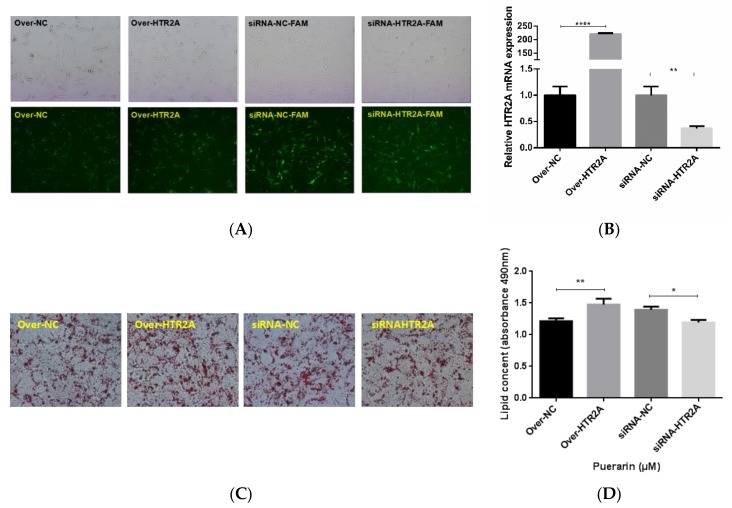
The role of HTR2A in the regulation of adipogenesis. (**A**): detection of transfection efficiency of HTR2A overexpression and knockdown (200×); (**B**): the expression level of HTR2A after HTR2A overexpression and knockdown; (**C**): oil Red O staining of induced differentiated adipocytes (200×); (**D**): the detection of lipid droplets of induced differentiated adipocytes. The panels in all of the figures were overexpression negative control, overexpression, knockdown negative control, and knockdown group from left to right. Values are means ± SD. vs. control group, * *p* < 0.05; ** *p* < 0.01; **** *p* < 0.001.

**Figure 7:**
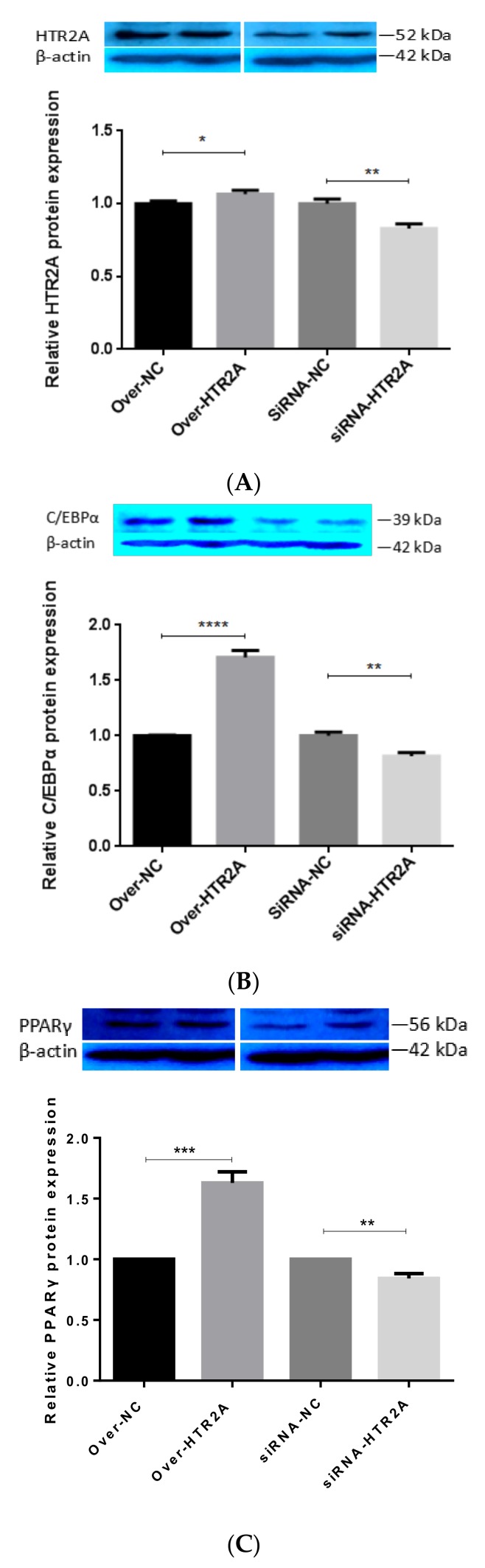
Regulation of adipogenesis markers by HTR2A. (**A**) The detection of HTR2A expression; (**B**) the detection of C/EBPα expression; (**C**) the detection of PPARγ expression. The lanes in all of the Western blots were overexpression negative control, overexpression, knockdown negative control, and knockdown group from left to right. Values are means ± SD. vs. control group, ** *p* < 0.01; *** *p* < 0.001 and **** *p* < 0.0001.

Previous studies have shown that HTR2A agonist could increase lipid accumulation and 5-HT suppress lipolysis in 3T3-L1 adipocytes and a genetic variant in HTR2A associated with obesity [6,7]. In this research, HTR2A was upregulated in Day-4 and Day-9 compared to Day-0, so it was chosen for functional analyses. Using the siRNA knockdown and overexpression technique, we examined the role of HTR2A on the pre-adipocytic differentiation of Yan Yellow cattle (Figure 6A). Compared with negative groups, the expression levels of HTR2A mRNA were significantly changed after the pre-adipocytes were transfected (*p* < 0.01) (Figure 6B). To determine the effects of HTR2A gene silencing or the over-expression of pre-adipocytic differentiation, pre-adipocytes were transfected with siRNA-HTR2A or over-HTR2A. The accumulated lipid droplet was measured by Oil Red O staining at Day-9 (Figure 6C). The results revealed that the formation of lipid droplets in the adipocytes was significantly increased in the over-HTR2A transfected group and decreased in the siRNA-HTR2A transfected group compared to the negative control group. Compared with cells from the negative control groups, the lipid content of the over-HTR2A transfected group significantly increased (Figure 6D). Meanwhile, the expression of the adipogenesis-related transcription factors C/EBPα and PPARγ increased in the over-HTR2A group (*p* < 0.0001), but decreased in the siRNA-HTR2A transfected group (*p* < 0.001) compared to the expression of them in the control groups (Figure 7).

### 2.6. The Effect of HTR2A in the Regulation of Adipogenesis by the Akt Signaling Pathway

**Figure 8:**
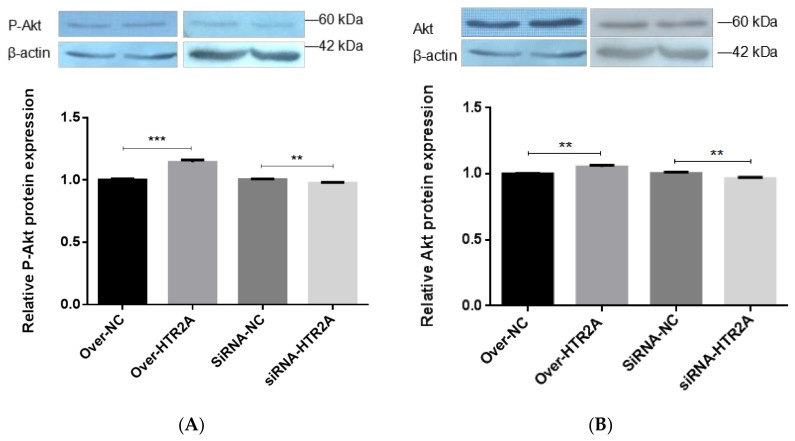
HTR5A affects Akt protein expression and phosphorylation. (**A**) The protein expression levels of phospho-AKT (Ser473) were evaluated after introduction; (**B**) the protein expression levels of Akt was evaluated after introduction; the level of total β-actin was determined as a loading control. The lanes in all of the Western blots were overexpression control, overexpression, knockdown control, and knockdown group from left to right. Values are means ± SD. vs. control group, ** *p* < 0.01; *** *p* < 0.001.

The PI3K-Akt signaling pathway is an important pathway involved in the differentiation of adipocytes. We explored the effects of HTR2A knockdown or overexpression on Akt phosphorylation during adipocyte differentiation. To determine the effects of HTR2A gene silencing or the over-expression of pre-adipocyte differentiation, pre-adipocytes were transfected with siRNA-HTR2A or over-HTR2A. Compared with the negative control group, the over-expression of HTR2A significantly activated the protein levels of phospho-AKT (Ser473), which represented activated phosphorylation of PI3K-Akt (*p* < 0.001) (Figure 8A). Furthermore, we observed a suppression of phospho-AKT (Ser473) in the siRNA-HTR2A transfected group relative to the negative control group (*p* < 0.01) (Figure 8A). Meanwhile, the expression level of Akt significantly increased in the over-HTR2A transfected group (*p* < 0.01), and the expression of Akt significantly decreased in the siRNA-HTR2A transfected group was observed (*p* < 0.01) (Figure 8B). These results indicated the effect of the regulation of the PI3K-Akt signaling pathway by HTR2A on adipogenesis.

## 3. Discussion

In recent years, Yan Yellow cattle, one of five local breeds of cattle in China, have become increasingly popular because of their excellent meat quality: for example, unique taste and typically high marbling pattern. However, the related fleshy traits of Yan Yellow cattle are oftentimes considered inferior compared to Wagyu and Hanwoo cattle. Therefore, understanding the molecular regulation mechanism and function of adipogenesis in Yan Yellow cattle can lead to successfully improving their meat quality. To investigate transcriptional information during adipocytic differentiation, we performed RNA-Seq at preadipocytes (Day-0), mid-differentiation stage (Day-4), and mature adipocytes (Day-9). 

By using GO classification followed by co-expression analyses, we obtained the genes most associated with biological processes, molecular function, and adipocytic differentiation, and predicted which genes are mainly responsible for adipogenesis in cattle. Several unigenes were assigned to chromosomes and chromosomal parts at the early stage, while at late stages unigenes were mainly distributed into the intrinsic components of plasma membranes in the cellular component categories. This indicated that growth-inhibited preadipocytes entered the cell cycle synchronously after receiving the appropriate mitogenic and adipogenic signals, underwent several cell divisions, and performed mitotic clonal expansion, all of which are a prerequisite for the terminal differentiation of adipocytes. After differentiation is initiated, preadipocytes can retain the ability to de-differentiate and re-enter mitosis but will proceed to terminal differentiation once they exit the DNA pre-synthesis phase [17,18,19,20]. Molecular functional enrichment analysis shows that differentially expressed genes are involved in several biological processes, including receptor activity, receptor binding, molecular transducer activity, and molecular function regulator. Remarkably, several DEGs are significantly enriched in pathways relating to cancer, the PI3K−Akt signaling pathway, cell cycle, the PPAR signaling pathway, and other biological processes, which suggest that different stages of adipogenesis may play different biological functions in receptor binding, receptor activity, and other aspects. In the present study, several DEGs were found between precursor adipocytes and mature adipocytes, which reflected the transcriptome characteristics of adipocytes at different stages. The functional enrichment of differentially expressed genes revealed that some DEGs were involved in adipogenesis. We obtained several adipogenesis-associated genes from the DEGs. mRNA expression of ABCA10, LPL, PLA2R1, FABP5, and HTR2A increased in the early stage, but was down-regulated in the terminal stage of adipocytic differentiation. While INSIG1 and FABP7 gradually decreased with the progress of adipogenesis, after analyzing the sequencing results in the different stages, we speculated that transcription regulatory regulators play a more important role in the early stages of adipogenesis. We evaluated the gene function of HTR2A by RNAi and overexpression techniques. RNA transient interference technology can temporarily interfere with the expression of RNA, thereby reducing gene expression of early stages of adipogenesis.

Additionally, we examined whether differentially expressed HTR2A can regulate adipogenesis. Serotonin, (5-hydroxytryptamine (5-HT), a neurotransmitter in the central and peripheral nervous systems, also appears to regulate energy homeostasis and glucose homeostasis (Reynolds et al., 2005). Serotonin receptor subtypes contain seven serotonin receptor families, known as 5-HT1R to 5-HT7R [21]. Several studies indicate that serotonin receptor 2A (5HT2A) is involved in the regulation of appetite and energy homeostasis [22,23]. 5HT2A-1438 G/A has been linked to several neuro-psychiatric disorders [17,24,25] and abdominal obesity [26,27]. Many serotonin receptor families have been reported to regulate adipogenesis. Previous studies have shown that serotonin receptors can mediate glycogen synthesis of hepatocytes and participate in the regulation of hepatic glucose metabolism [5]. HTR2A agonist treatment increased lipid accumulation and 5-HT suppressed lipolysis using 3T3-L1 adipocytes [6]. Based on RNA-Seq, HTR2A was upregulated significantly at Day-4 and decreased slightly at Day-9 as a positive regulator. The expression of HTR2A by qPCR was consistent with the RNA-Seq, suggesting that HTR2A may be a start-up function for adipogenesis. We then used RNAi techniques to interfere with its expression, the results showed that the RNAi did slightly reduce expression of adipogenesis marker mRNA and lipid accumulation in adipocytes. In contrast, overexpression of HTR2A promoted adipogenesis, which demonstrated that HTR2A is a positive regulator of adipogenesis. Subsequent analyses of the causes of this phenomenon suggested that the HTR2A gene can affect the formation of lipid droplets because these genes play an important role in preadipocyte differentiation. According to the results of RNA-seq, we hypothesized that the regulation effect of HTR2A on adipogenesis may be related to the PI3K/Akt signaling pathway. Finally, we tested the expression of the AKt signaling pathway in subsequent experiments.

The PI3K/Akt signaling pathway is a classic insulin signaling pathway [28]. Akt, known as PKB or Rac, plays a key role in the regulation of cell growth and apoptosis. Upon activation by extracellular signaling molecules necessary for insulin and various growth stages and cell survival, PI3 kinase plays a role in inhibitor-sensitive pathways [29,30]. Another important function of Akt is to regulate glycogen synthesis by the phosphorylation and inactivation of GSK-3 alpha and beta and regulating insulin-stimulated glucose transportation [31,32]. PI3K signaling is involved in the regulation of several cell functions, such as proliferation, differentiation, apoptosis, and glucose transport. Several studies have shown that activation of the Akt pathway regulates the expression of PPARγ and C/EBPα during adipogenesis and can promote or inhibit the adipogenic differentiation of adipocytes [33,34,35,36,37].

Accordingly, these findings indicate that the phosphorylation of phospho-AKT (Ser473) is suppressed in the Akt signaling pathway following silencing of the HTR2A gene, which can reduce the activity of Akt. As signaling pathway phosphorylation of phospho-AKT (Ser473) plays an important role in proliferation, growth, and differentiation of adipocytes, the regulation of signaling pathway phosphorylation might regulate adipogenesis after silencing and overexpression of the HTR2A gene, which demonstrated that HTR2A is a regulator of adipogenesis. The results showed that HTR2A was most likely to regulate adipogenesis by phosphorylation of the Akt signaling pathway as a potential regulatory factor.

In conclusion, this is a novel study comparing the DEGs of adipocytes during adipogenic differentiation in Yan Yellow cattle. RNA-Seq analysis identified HTR2A as a potential regulatory factor that can regulate adipogenesis by phosphorylation of the Akt signaling pathway. Our study generated useful genetic resources and sequencing information of considerable value for further research on Yan Yellow cattle. Functional characterization of several unigenes involved in the identified biological processes could stimulate research to further understand the molecular basis of adipose differentiation in Yan Yellow cattle, and also provide a reference base for promoting intramuscular fat deposition.

## 4. Materials and Methods

### 4.1. Ethics Statement

This research protocol was considered and approved by the Animal Welfare and Ethics Committee of Jilin Academy of Agricultural Sciences (AWEC2017A01, 9 March 2017). The cattle were handled in accordance with good animal practice as required by the Animal Ethics Procedures and Guidelines of the People's Republic of China.

### 4.2. Isolation and Identification of Preadipocytes

The preadipocytes were extracted from dorsal subcutaneous fetal bovine adipose tissue. After cutting the fatty tissues into pieces with volumes of approximately 1 mm^3^ by scissors, they were digested by collagenase Ι (Sangon Biotech, Shanghai, China) and seeded into plates at a density of 6 × 10^5^ cells/plate. When the cells reached confluence (Day-0), they were induced by differentiation medium Ι (10 μg/mL insulin, 0.5 mM 1-methyl-3-isobutylxanthine, 1.0 μM dexamethasone) (Sigma, Shanghai, China) for 48 h. Next, the cells were cultured with Dulbecco’s Modified Eagle’s medium(DMEM) (Sangon Biotech) containing 10 μg/mL insulin (differentiation medium II) for an additional 48 h (Day-4) and further cultured in DMEM without insulin until day 9 (Day-9). The accumulation of lipid droplets, content of triacylglycerol (TG), and mRNA expression levels of adipogenesis-related transcription factors were measured by staining of Oil Red O (Sangon Biotech), Triglyceride assay kit (Applygen Technology Inc., Beijing, China) and RT-qPCR, respectively.

### 4.3. RNA-Seq Library Preparation and Sequencing Analysis

Total RNA was extracted with TRIzol reagent (Invitrogen, Beijing, China) according to manufacturer protocols. The Quantity and purity of the RNA were detected using a Nanodrop 2000C (ThermoFisher scientific, Wilmington, DE, USA) and a Qubit Fluorometer (Invirogen, Carlsbad, CA, USA). The RNA was stored at −80 °C until further analyses. Library construction was performed according to the Illumina sample preparation for RNA-seq protocol. The mRNA of eukaryotic cells was enriched by magnetic beads with Oligo (dT) after the samples were qualified. When the enrichment was complete, the mRNA was interrupted into short segments with the addition of a fragmentation buffer. Subsequently, double-stranded cDNA was synthesized by reverse transcription using 6-base random primers. The purified double-stranded cDNA was subjected to terminal reparation, singe nucleotide A (Adenine) addition, and serial sequencing. The fragment size of double-stranded cDNA was selected by an AMpure XP bead (Beckman coulter, Shanghai, China), and the selected double-stranded cDNA was subjected to PCR enrichment to construct a cDNA library. Constructing and sequencing the RNA-seq library for each sample was conducted by Beijing Compass Bio-Technology Co., Ltd. China (www.kangpusen.com) based on the protocols of Illumina HiSeqTM2500/MiSeq™ to generate paired-end reads (150 bp in length). The quality of RNA-seq reads from all the adipose tissues was checked using FastQC (0.11.5, Babraham institute, Cambridge, UK). The reads which passed the quality control were mapped to the Bos Taurus genome (BosTau6 UMD3.1.89) from Ensembl using STAR (2.5.2a, Cold Spring Harbor Laboratory, Cold Spring Harbor, NY, USA).

### 4.4. Statistical Analysis and DEGs Identification

The accuracy of the sample statistic RPKM (Reads per Kilobase of exon model per Million mapped reads) was affected by the depth of sample sequencing. In this case, the accuracy of the sample statistics was measured by a subsample or bootstrap with partial data. To determine the estimate gene expression, we subsampled a series of read sets from all reads to calculate the RPKM value of each set respectively. The abundance of transcription was the most direct indicator of gene expression level. RPKM was the most commonly used gene expression level measurement method, and can compare expression level during adipogenesis in different experimental conditions by RPKM gene distribution.

Differences in gene expression were analyzed by HTSeq v0.5.4p3. Read count data were standardized with TMM, and the significance and fold-change (*p* < 0.05 and log2 fold change > |1|) were set, the differences in expression were analyzed by DEGseq (Version 1.34.0). The overall distribution of the differential genes was showed by Volcano plot and Venn diagram. By aggregating identical or similar genes in expression patterns, the function of unknown genes or the unknown function of known genes was identified. DEGs were subjected to GO functional and KEGG Pathway analyses after setting the significance threshold (*p* < 0.05).

### 4.5. Quantitative Real-Time PCR

To validate the accuracy of DEGs, eight DE genes with known functions in adipogenesis and lipid metabolism were chosen as candidates for analysis by RT-qPCR. Regarding relative gene expression, β-actin was selected as a normalized internal control. Primer sequences for target genes are shown in Table 4. Reaction mixtures were incubated for pre-denaturation at 95 °C for 5 min, followed by 40 PCR cycles: 10 s at 95 °C, 15 s, for 60 °C and 20 s, for 72 °C. Relative expression was calculated using the 2^−ΔΔ*C*t^ method.

### 4.6. siRNA Knockdown and Overexpression Plasmid DNA Transfection

siRNA knockdown and overexpression recombinant plasmids (pGPU6/GFP/Neo-HTR2A and pEX/EGFP-4-HTR2A) used in this experiment were purchased from GenePharma (Shanghai, China). After toxicity was evaluated by empty plasmid, 2 μg recombinant plasmids were transfected into preadipocytes with FuGENETM HD Transfection Reagent (Promega Corporation, Madison, WI, USA). The transfected cells were induced in differentiation medium I for 2 days after transfection.

### 4.7. Western Blot

Adipocytes were lysed by RIPA Lysis and Extraction Buffer (ThermoFisher Technology, Beijing, China) containing Protease and Phosphatase Inhibitor Cocktail (ThermoFisher Technology). Protein content was determined by BCA protein assay reagent (Betotime Biotechnology, Shanghai, China). PVDF membranes were incubated with the following antibodies: Akt (Cell Signaling Technology, Shanghai, China), Phospho-Akt (Ser473) (Cell Signaling Technology), PPARγ (Abcam, Shanghai, China), C/EBPα (Absin Bioscience, Shanghai, China), and Anti-β-actin (Abcam) at a temperature of 4 °C overnight, followed by incubation with Anti-mouse IgG HRP-linked antibody (Cell Signaling Technology) or Anti-rabbit IgG HRP-linked antibody (Cell Signaling Technology) at room temperature for 1 h. Immunoreactive proteins were detected by Super ECL Plus system (Applygen Technology Inc., Beijing, China) and quantified using Image J software (1.8.0_112, National Institutes of Health, Bethesda, MD, USA).

### 4.8. Statistical Analysis

All data were expressed as the mean ± SD. For all statistical analyses, *p* < 0.05 was considered significant. One-way ANOVA and post hoc test were conducted for all statistical significance analysis by GraphPad Prism software (graphpad prism 7, GraphPad Prism software, Inc., La Jolla, CA, USA). Each mean value was obtained from three independent experiments.

## Figures and Tables

**Table 1:** Quality of Sequencing.

Sample	Raw Reads	Clean Reads	Clean Base (G)	Q20 (%)	GC Content (%)
Day-0	22,082,379	21,842,223	6.55	93.45	51.52
Day-4	24,642,129	24,327,149	7.29	93.5	51.77
Day-9	22,976,664	22,729,161	6.82	93.83	52.33

**Table 2:** Statistics and mapping results of RNA-Seq data.

Sample	Clean Reads	Uniquely Mapped Reads Number	Number of Reads Mapped to Multiple Loci	Number of Reads Mapped to Too Many LOCI
Day-0	21,842,223	19,901,042 (91.11%)	520,263 (2.38%)	27,865 (0.13%)
Day-4	24,327,149	22,285,077 (91.61%)	512,727 (2.11%)	24,311 (0.10%)
Day-9	22,729,161	20,700,769 (91.08%)	480,052 (2.11%)	19,016 (0.08%)

**Table 3:** Summary of pathways associated with DEGs between early and late stage groups using KEGG pathway databases.

Category	Groups	Gene Count	Genes	*p* Value
Extracellular matrixc(ECM)–receptor interaction	Day-0 vs. Day-4	18	FN1/THBS1/LAMB1/LAMA4/HSPG2/LAMC1/COL4A1/COL4A2/ITGB3/HMMR/LAMC2/ITGA10/LOC530102/AGRN/CHAD/ITGA2/LAMB3/SV2C	8.72 × 10^−12^
	Day-4 vs. Day-9	4	TNC/COL6A1/COL6A2/TNN	4.74 × 10^−^^4^
PI3K-Akt signaling pathway	Day-0 vs. Day-4	30	FN1/THBS1/LAMB1/LAMA4/LAMC1/COL4A1/COL4A2/YWHAQ/MET/ITGB3/CDK4/PKN1/LAMC2/BRCA1/CREB3L2/ITGA10/NGFR/PHLPP1/LOC530102/CCNE2/MYB/HGF/TEK/CHAD/ITGA2/LAMB3/PIK3CD/EFNA5/FGF9/GNG4	9.93 × 10^−^^8^
	Day-4 vs. Day-9	17	TNC/COL6A1/COL6A2/GYS1/PCK2/TNN/ANGPT4/INSR/PPP2R3C/KITLG/TLR2/PDGFA/IL7/FGF1/IFNB1/GNG3/PPP2R2B	6.62 × 10^−^^5^
Pathways in cancer	Day-0 vs. Day-4	33	FN1/LAMB1/LAMA4/LAMC1/COL4A1/COL4A2/FZD1/MET/BIRC5/CDK4/EGLN3/CKS1B/TGFB2/RUNX1/RAD51/LAMC2/WNT11/E2F1/CCNE2/HGF/SUFU/PLCB1/ITGA2/LAMB3/CYCS/LOC100847700/PIK3CD/FGF9/PLCB4/FZD5/EDNRB/CDH1/GNG4	1.74 × 10^−^^7^
	Day-4 vs. Day-9	19	ABL1/PTGS2/JUN/STAT1/CXCL8/PPARG/NFKBIA/KITLG/MITF/NOS2/FLT3LG/FZD4/PDGFA/FGF1/WNT9A/GLI2/LEF1/FZD9/GNG3	1.32 × 10^−^^4^
Focal adhesion	Day-0 vs. Day-4	22	FN1/THBS1/LAMB1/LAMA4/LAMC1/COL4A1/COL4A2/MET/ITGA8/FYN/ITGB3//ITGA3/LAMC2/ITGA10/LOC530102//HGF/CHAD/PARVB/ITGA2/LAMB3/PIK3CD/RASGRF1	1.64 × 10^−^^6^
	Day-4 vs. Day-9	7	TNC/COL6A1/COL6A2/CCNDNN/ELK1/MYLK2/PDGFA	1.74 × 10^−^^3^
Axon guidance	Day-0 vs. Day-4	14	MET/EPHA4/RND1/TRPC4/PLCG2/ROBO2/SEMA3B/ROBO3/SEMA6B/NTNG1/PIK3CD/EFNA5/NTN3/BMP7	6.40 × 10^−^^6^
	Day-4 vs. Day-9	8	ABL1/EPHB3/L1CAM/SEMA7A/SEMA3E/CDK5/EPHA1/PLCG2	2.30 × 10^−^^4^

**Table 4:** Primer sequences for target genes.

Primer	Forward	Reverse
ABCA10	CGCCCAAGAAACGACTC	GAAAAGCCACAAACCCG
INSIG1	AGAGCCACACAAGTTCAAGC	AGCCAGGAGCGGATGTAGAG
LPL	AGGACACTTGCCACCTCATT	CATCCGCCATCCAGTTCATA
PLA2R1	GCCGATACGAAAGAGACG	CCAAGTGCTTCCTTACGA
FABP5	GAAGGAAGTAGGAGTGGGGA	CTGTGTTGTTTTCAAAGTGC
PPARγ	CGGAAGCCCTTTGGTGACTTTATG	GCAGCAGGTTGTCTTGGATGTC
FABP7	AGTCTGTTGTTAGCCTGG	CAGGTCAGGATGTTTTCT
HTR2A	GACCGTGGACTCAGAAAATC	TCAGGAAATAGTTGGTGGCA
β-actin	AGATCAAGATCATCGCGCCC	TAACGCAGCTAACAGTCCGC

## Supplementary Materials

The following are available online at http://www.mdpi.com/1422-0067/19/6/1760/s1.

## Abbreviations

DEGs	differentially expressed genes
C/EBPα	CCAAT-enhancer-binding protein α
PPARγ	peroxisome proliferator-activated receptor γ
HTR2A	5-hydroxytryptamine receptor 2A
IMF	intramuscular fat
RNA-seq	RNA sequencing
GO	Gene Ontology
KEGG	Kyoto Encyclopedia of Genes and genomes
RPKM	Reads Per Kilobase of exon model per Million mapped reads

## References

[1] Alessi M.C., Peiretti F., Morange P., Henry M., Nalbone G., Juhan-Vague I. (1997). Production of plasminogen activator inhibitor 1 by human adipose tissue: Possible link between visceral fat accumulation and vascular disease. Diabetes.

[2] Avram M.M., Avram A.S., James W.D. (2007). Subcutaneous fat in normal and diseased states 3. Adipogenesis: From stem cell to fat cell. J. Am. Acad. Dermatol..

[3] Finelli C., Sommella L., Gioia S., La Sala N., Tarantino G. (2013). Should visceral fat be reduced to increase longevity?. Ageing Res. Rev..

[4] Marston O.J., Garfield A.S., Heisler L.K. (2011). Role of central serotonin and melanocortin systems in the control of energy balance. Eur. J. Pharmacol..

[5] Tudhope S.J., Wang C.C., Petrie J.L., Potts L., Malcomson F., Kieswich J., Yaqoob M.M., Arden C., Hampson L.J., Agius L. (2012). A novel mechanism for regulating hepatic glycogen synthesis involving serotonin and cyclin-dependent kinase-5. Diabetes.

[6] Oh C.M., Namkung J., Go Y., Shong K.E., Kim K., Kim H., Park B.Y., Lee H.W., Jeon Y.H., Song J. (2015). Regulation of systemic energy homeostasis by serotonin in adipose tissues. Nat. Commun..

[7] Zhao H., Wilkinson A., Shen J., Wu X., Chow W.H. (2017). Genetic polymorphisms in genes related to risk-taking behaviours predicting body mass index trajectory among Mexican American adolescents. Pediatr. Obes..

[8] Margawati E.T. (2012). A global strategy of using molecular genetic information to improve genetics in livestock. Reprod. Domest. Anim..

[9] Goessling W., North T.E., Loewer S., Lord A.M., Lee S., Stoick-Cooper C.L., Weidinger G., Puder M., Daley G.Q., Moon R.T. (2009). Genetic interaction of PGE2 and Wnt signaling regulates developmental specification of stem cells and regeneration. Cell.

[10] Robelin J. (1981). Cellularity of bovine adipose tissues: Developmental changes from 15 to 65 percent mature weight. J. Lipid Res..

[11] Cianzio D.S., Topel D.G., Whitehurst G.B., Beitz D.C., Self H.L. (1985). Adipose tissue growth and cellularity: Changes in bovine adipocyte size and number. J. Anim. Sci..

[12] Du M., Yin J., Zhu M.J. (2010). Cellular signaling pathways regulating the initial stage of adipogenesis and marbling of skeletal muscle. Meat Sci..

[13] Basu U., Romao J.M., Guan L.L. (2013). Adipogenic transcriptome profiling using high throughput technologies. J. Genom..

[14] Wang Y.H., Bower N.I., Reverter A., Tan S.H., De Jager N., Wang R., McWilliam S.M., Cafe L.M., Greenwood P.L., Lehnert S.A. (2009). Gene expression patterns during intramuscular fat development in cattle. J. Anim. Sci..

[15] Jin W., Olson E.N., Moore S.S., Basarab J.A., Basu U., Guan L.L. (2012). Transcriptome analysis of subcutaneous adipose tissues in beef cattle using 3′ digital gene expression-tag profiling. J. Anim. Sci..

[16] Lee H.J., Jang M., Kim H., Kwak W., Park W., Hwang J.Y., Lee C.K., Jang G.W., Park M.N., Kim H.C. (2013). Comparative transcriptome analysis of adipose tissues reveals that ECM-receptor interaction is involved in the depot-specific adipogenesis in cattle. PLoS ONE..

[17] Pinborg L.H., Arfan H., Haugbol S., Kyvik K.O., Hjelmborg J.V., Svarer C., Frokjaer V.G., Paulson O.B., Holm S., Knudsen G.M. (2008). The 5-HT2A receptor binding pattern in the human brain is strongly genetically determined. NeuroImage.

[18] Yeh W.C., Bierer B.E., McKnight S.L. (1995). Rapamycin inhibits clonal expansion and adipogenic differentiation of 3T3-L1 cells. Proc. Natl. Acad. Sci. USA.

[19] Reichert M., Eick D. (1999). Analysis of cell cycle arrest in adipocyte differentiation. Oncogene.

[20] Tang Q.Q., Otto T.C., Lane M.D. (2003). Mitotic clonal expansion: A synchronous process required for adipogenesis. Proc. Natl. Acad. Sci. USA.

[21] Lam D.D., Heisler L.K. (2007). Serotonin and energy balance: Molecular mechanisms and implications for type 2 diabetes. Expert Rev. Mol. Med..

[22] Reynolds G.P., Templeman L.A., Zhang Z.J. (2005). The role of 5-HT2C receptor polymorphisms in the pharmacogenetics of antipsychotic drug treatment. Prog. Neuro-Psychopharmacol. Biol. Psychiatry.

[23] Huang X.F., Han M., Storlien L.H. (2004). Differential expression of 5-HT(2A) and 5-HT(2C) receptor mRNAs in mice prone, or resistant, to chronic high-fat dietinduced obesity. Mol. Brain Res..

[24] Meyer J.H., McMain S., Kennedy S.H., Korman L., Brown G.M., DaSilva J.N., Wilson A.A., Blak T., Eynan-Harvey R., Goulding V.S. (2003). Dysfunctional Attitudes and 5-HT2 Receptors During Depression and Self-Harm. Am. J. Psychiatry.

[25] Messa C., Colombo C., Moresco R.M., Gobbo C., Galli L., Lucignani G., Gilardi M.C., Rizzo G., Smeraldi E., Zanardi R., Artigas F., Fazio F. (2003). 5-HT2A receptor binding is reduced in drug-naive and unchanged in SSRI responder depressed patients compared to healthy controls: A PET study. Psychopharmacology (Berl.).

[26] Rosmond R., Bouchard C., Bjorntorp P. (2002). 5-HT2A receptor gene promoter polymorphism in relation to abdominal obesity and cortisol. Obes. Res..

[27] Rosmond R., Bouchard C., Bjorntorp P. (2002). Increased Abdominal Obesity in Subjects with a Mutation in the 5-HT2A Receptor Gene Promoter. Ann. N. Y. Acad. Sci..

[28] Sahiel A.R., Pessin J.E. (2000). Signaling pathways in insulin action: Molecular targets of insulin resistance. J. Clin. Invest..

[29] Franke T.F., Kaplan D.R., Cantley L.C. (1997). PI3K: Downstream AKTion blocks apoptosis. Cell.

[30] Burgering B.M., Coffer P.J. (1995). Protein kinase B (c-Akt) in phosphatidylinositol-3-OH kinase signal transduction. Nature.

[31] Hajduch E., Litherland G.J., Hundal H.S. (2001). Protein kinase B (PKB/Akt)—A key regulator of glucose transport?. FEBS Lett..

[32] Cross D.A., Alessi D.R., Cohen P., Andjelkovich M., Hemmings B.A. (1995). Inhibition of glycogen synthase kinase-3 by insulin mediated by protein kinase, B. Nature.

[33] Peng X.D., Xu P.Z., Chen M.L., Hahn-Windgassen A., Skeen J., Jacobs J., Sundararajan D., Chen W.S., Crawford S.E., Coleman K.G. (2003). Dwarfism, impaired skin development, skeletal muscle atrophy, delayed bone development, and impeded adipogenesis in mice lacking Akt1 and Akt2. Genes Dev..

[34] Kim S.P., Ha J.M., Yun S.J., Kim E.K., Chung S.W., Hong K.W., Kim C.D., Bae S.S. (2010). Transcriptional activation of peroxisome proliferator- activated receptor-gamma requires activation of both protein kinase A and Akt during adipocyte differentiation. Biochem. Biophys. Res. Commun..

[35] Smith P.J., Wise L.S., Berkowitz R., Wan C., Rubin C.S. (1988). Insulin-like growth factor-I is an essential regulator of the differentiation of 3T3-L1 adipocytes. J. Biol. Chem..

[36] Cornelius P., MacDougald O.A., Lane M.D. (1994). Regulation of adipocyte development. Annu. Rev. Nutr..

[37] Kohn A.D., Summers S.A., Birnbaum M.J., Roth R.A. (1996). Expression of a constitutively active Akt Ser/Thr kinase in 3T3-L1 adipocytes stimulates glucose uptake and glucose transporter 4 translocation. J. Biol. Chem..

