# Supplementary material for: RNA-Seq Analysis Reveals a Positive Role of HTR2A in Adipogenesis in Yan Yellow Cattle
Source: Int J Mol Sci. 2018 Jun 13;19(6):1760. doi: 10.3390/ijms19061760 (PMC6032390; doi:10.3390/ijms19061760)
Supplement: Supplementary file 1 [file ijms-19-01760-s001.pdf]

Supplementary Materials

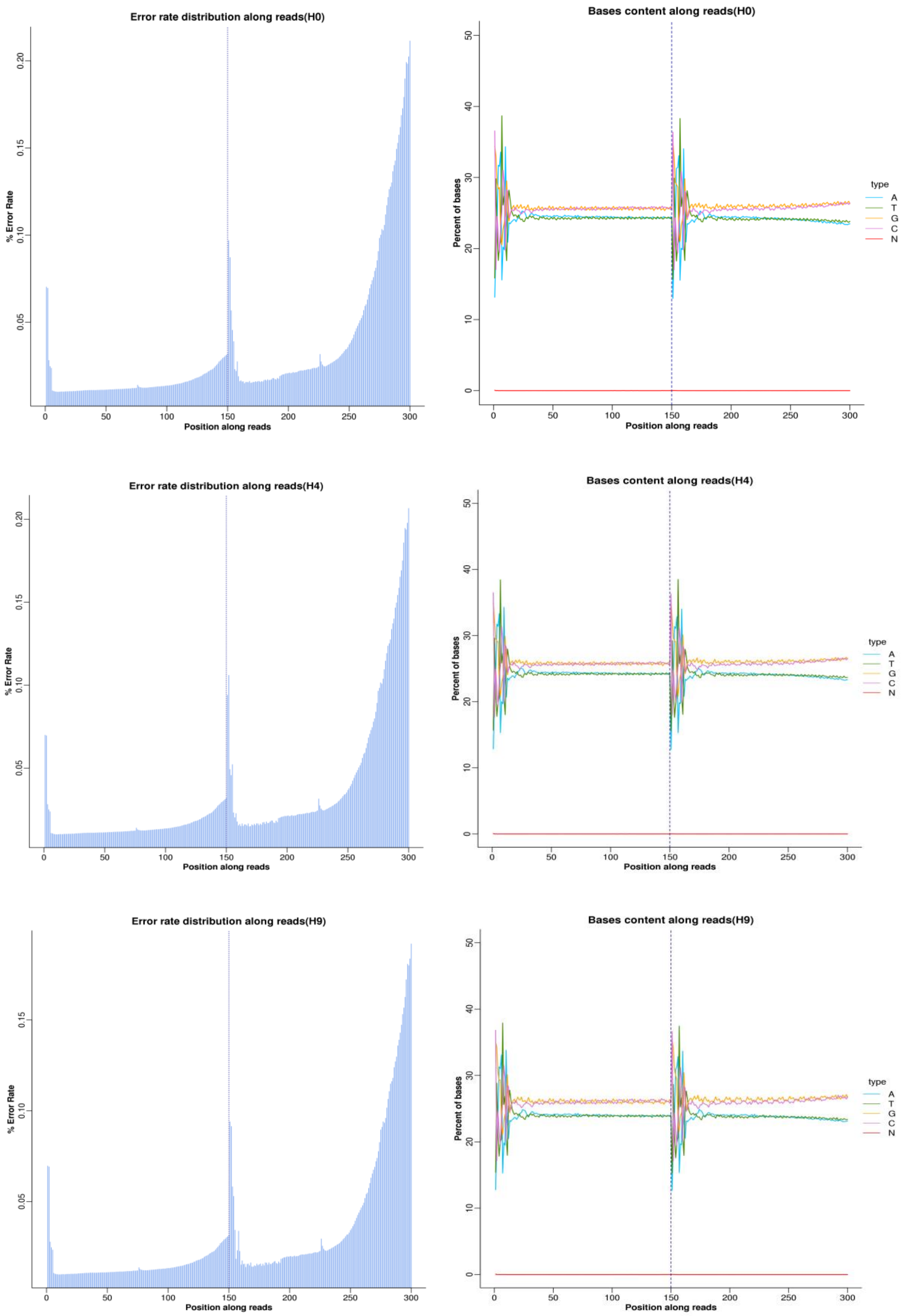

A

B

C

Figure S1. Comparison of error rate distribution and bases content along reads during varieties of adipogenesis stages. (**A**): The Q20 % and the GC concent (%) of early stage of differentiation (Day-0); (**B**): The Q20 % and the GC concent (%) of middle stage of differentiation (Day-4); (**C**): The Q20 % and

the GC concent (%) of mature stage of differentiation (Day-9). Error identification ratio of nucleotides with Phred quality score < 0.01.
